# ACE inhibition attenuates uremia-induced aortic valve thickening in a novel mouse model

**DOI:** 10.1186/1471-2261-9-10

**Published:** 2009-03-03

**Authors:** Mikko A Simolin, Tanja X Pedersen, Susanne Bro, Mikko I Mäyränpää, Satu Helske, Lars B Nielsen, Petri T Kovanen

**Affiliations:** 1 Wihuri Research Institute, Helsinki, Finland; 2 Department of Clinical Biochemistry, Rigshospitalet, University of Copenhagen, Copenhagen, Denmark; 3 Department of Nephrology, Rigshospitalet, University of Copenhagen, Copenhagen, Denmark; 4 Department of Biomedical Sciences, University of Copenhagen, Copenhagen, Denmark

## Abstract

**Background:**

We examined whether impaired renal function causes thickening of the aortic valve leaflets in hyperlipidemic apoE-knockout (apoE^-/-^) mice, and whether the putative effect on the aortic valves could be prevented by inhibiting the angiotensin-converting enzyme (ACE) with enalapril.

**Methods:**

Thickening of the aortic valve leaflets in apoE^-/- ^mice was induced by producing mild or moderate chronic renal failure resulting from unilateral nephrectomy (1/2 NX, n = 18) or subtotal nephrectomy (5/6 NX, n = 22), respectively. Additionally, the 5/6 NX mice were randomized to no treatment (n = 8) or enalapril treatment (n = 13). The maximal thickness of each leaflet was measured from histological sections of the aortic roots.

**Results:**

Leaflet thickness was significantly greater in the 5/6 NX mice than in the 1/2 NX mice (P = 0.030) or the unoperated mice (P = 0.003). The 5/6 NX mice treated with enalapril had significantly thinner leaflets than did the untreated 5/6 NX mice (P = 0.014).

**Conclusion:**

Moderate uremia causes thickening of the aortic valves in apoE^-/- ^mice, which can be attenuated by ACE inhibition. The nephrectomized apoE^-/- ^mouse constitutes a new model for investigating the mechanisms of uremia-induced aortic valve disease, and also provides an opportunity to study its pharmacologic prevention.

## Background

The prevalence of cardiovascular disease (CVD) is far greater in patients with chronic renal failure (CRF) than in the general population [1]. Similarly, the progression of aortic stenosis (AS) is accelerated in chronically uremic patients [2-4]. In addition to renal failure, epidemiological studies have identified that other atherosclerotic risk factors, including hypertension, smoking, hypercholesterolemia, diabetes mellitus, male gender, and age, predispose to AS development [5-7]. Stenotic aortic valves are characterized by an atherosclerosis-like lesion, consisting of inflammatory cells, cholesterol deposits, and calcified nodules [8]. Furthermore, active bone formation and adverse extracellular matrix remodeling contribute to the thickening and stiffening of the valves [9-11]. Importantly, angiotensin-converting enzyme (ACE), angiotensin II (Ang II), and Ang II type 1 receptors (AT-1Rs) are expressed in the aortic valves and ACE activity is augmented in the stenotic valves [12,13], suggesting that inappropriate activation of the renin-angiotensin system (RAS) participates in the pathogenesis of AS. To fully understand the pathogenic mechanisms of AS and eventually to be able to design potential drug therapies, animal models suitable for preclinical studies of AS are needed. To date, several rabbit models of AS have been introduced [14-17]. In addition, different mouse models of AS have been tested. These include old apoE^-/- ^mice [18] and old LDLr^-/- ^apoB^100/100 ^mice [19], which are prone to develop functional valvular heart disease, and wild-type mice in which a high fat/high carbohydrate diet was shown to trigger thickening of the aortic valve leaflets [20].

In view of the higher prevalence of aortic valve calcification and stenosis among patients with renal failure, and the pathogenetic similarities between AS and atherosclerosis, we hypothesized that chronic uremia would cause aortic valve thickening, a condition known to ultimately lead to functional impairment and AS development. We chose the nephrectomized apoE^-/- ^mouse, which develops extensive vascular disease and is already an established animal model in the study of uremic atherosclerosis [21,22] and could thus serve as a suitable model of uremia-induced AS. Moreover, to learn whether Ang II-dependent pathways may contribute to uremia-associated aortic valve thickening, we tested the effect of enalapril, an ACE inhibitor, on valvular thickening in this particular mouse model.

## Methods

### Animals

**Figure 1:**
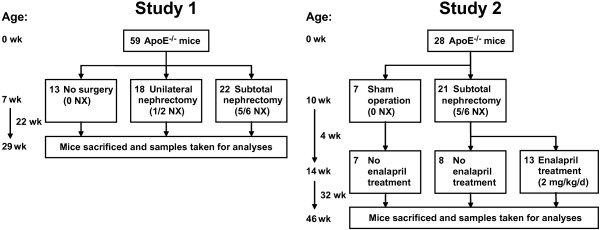
**Flowcharts showing the study designs in Study 1 and Study 2**.

We examined the valvular leaflets in hearts from mice that were also used to study the effects of uremia on the development of atherosclerosis [21,22]. The studies were performed according to the principles stated in the Danish law on animal experiments and approved by the Animal Experiments Inspectorate, Ministry of Justice, Denmark. The animals were male apoE^-/- ^mice (C57BL/6JBom-ApoE^tm1Unc^), which had been backcrossed 10 generations onto the C57BL/6 background (M&B Laboratory Animals and Services for Biomedical Research, Ry, Denmark) and fed with a standard mouse diet (Altromin 1314, Altromin, Lage, Germany). The investigation was divided into two substudies, which are referred to as Study 1 and Study 2 in the text. The study design of both studies is shown in Figure 1.

In Study 1, the mice were assigned at the age of seven weeks to one of three groups: (1) unoperated (0 NX, n = 13), (2) unilateral nephrectomy (1/2 NX, n = 18) in which the left kidney was removed, or (3) subtotal nephrectomy (5/6 NX, n = 22) in which both the left kidney and the upper and lower poles of the right kidney were resected. The surgery was performed under anesthesia with postoperative analgetics as previously described [21]. The mice were sacrificed 22 weeks after allocation to the different groups, and the portion of the heart containing the proximal ascending aorta was removed, fixed in formalin and embedded in paraffin [21].

In Study 2, the mice were allocated at the age of 10 weeks to either 5/6 NX or a sham operation (0 NX). In the 5/6 NX group, the upper and lower poles of the right kidney were resected, and two weeks later the entire left kidney was resected [22]. The sham-operated mice underwent kidney exposure operations at the same time points. To study the effect of ACE inhibition on the development of aortic valve thickening, treatment with an ACE inhibitor (2 mg/kg/d enalapril) was started four weeks after nephrectomy. After 36 weeks of uremia, the mice were sacrificed and the tissues were snap frozen, thawed, and fixed in formalin, after which they were embedded in Tissue-Tek OCT (Sakura Finetek Europe B.V., Zoeterwoude, The Netherlands) and frozen again [22].

Measurements of body weight, blood pressure, blood hemoglobin, the plasma levels of urea, creatinine, phosphate, calcium, cholesterol, and triglycerides were performed as previously described [21,22].

### Histology

**Figure 2:**
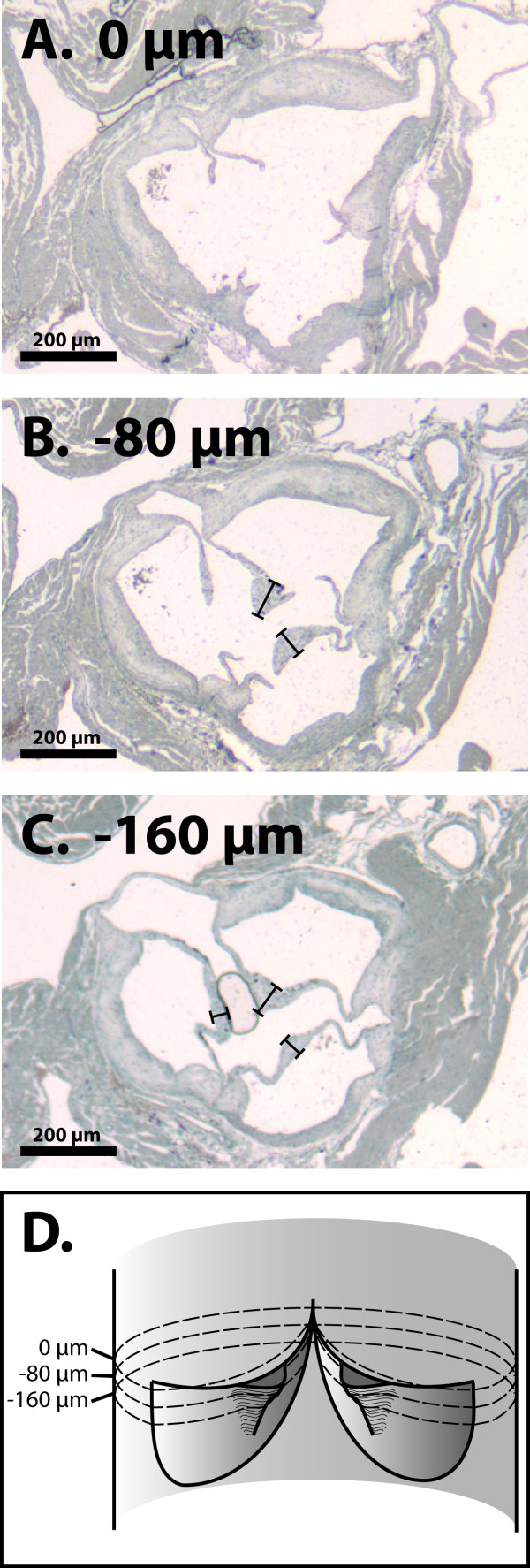
**Histological cross-sections of the aortic root showing three levels of cutting of the aortic valve**. The sections were chosen to show (A) the commissural level (0 μm), and the levels (B) -80 μm and (C) -160 μm towards the base of aortic valve. Note that at 0 μm, only the leaflet commissures are visible, demonstrating that the free edge of the leaflet is below the 0 μm level. The maximal leaflet thickness (measured at the nodule of Arantius located at the center of a leaflet) is smaller at -160 μm than at -80 μm, reflecting the gradual thickening of the nodule towards the free edge of the leaflet. The sections were collected starting from the base of the valve and moving towards the commissural level, which is defined as the reference level (0 μm) in this stack of sections. Panel (D) illustrates the cutting levels in the aortic root. The sample is derived from a uremic mouse (Study 1). Photographed using 4× magnification.

In Study 1, 4-μm-thick sections were cut from the paraffin-embedded blocks, beginning at the apex of the heart. Collection of the sections was started when leaflet commissures first appeared, and then continued to include the first 160 μm of the aortic root (see Figure 2A–C). In Study 2, 8-μm-thick frozen sections were cut, beginning at the apex of the heart. Collection of the sections was started once three leaflets became visible, and then continued to include the first 240 μm of the aortic root.

**Figure 3:**
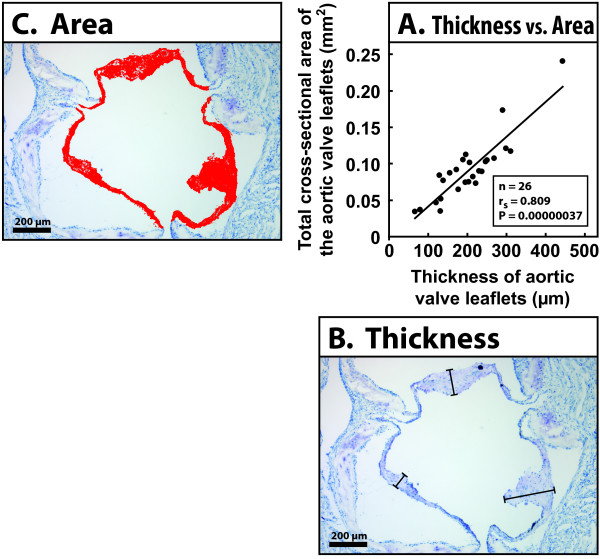
**Correlation between the thickness and total cross-sectional area of the aortic valve leaflets**. In the graphical presentation (A), each point denotes one mouse (Study 2). The thickness of the aortic valve leaflets is the average value of the three thickest leaflets in six sections per mouse. The total cross-sectional area of the aortic valve leaflets is the average value of three sections per mouse. Panels B and C show typical sections from which the measurements were made. In panel (B), each bar denotes the maximal thickness of a leaflet, and in panel (C) the red color shows the area measured to obtain the total area of the three leaflets in one section. N-value, Spearman's correlation coefficient, and P-value are shown.

**Figure 4:**
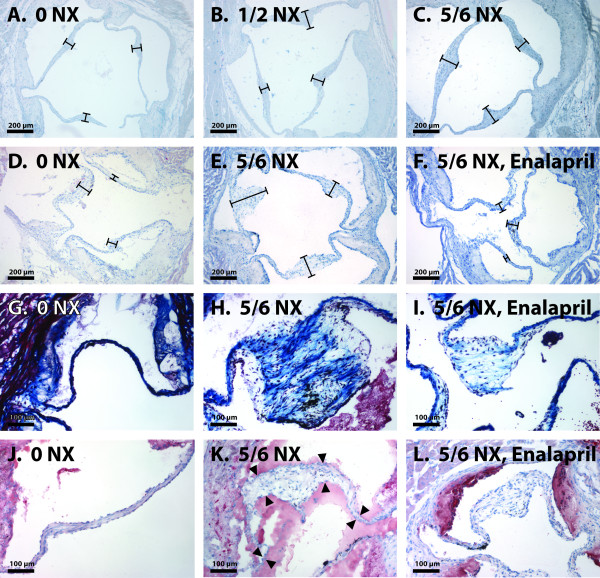
**Histological  sections showing valvular thickness, and stainings of the valves for the detection of fibrosis and neutral lipids in variously treated apoE^-/-^ mice**. Typical hematoxylin-stained sections from (A) unoperated controls (0 NX), (B) unilaterally nephrectomized (1/2 NX), and (C) subtotally nephrectomized (5/6 NX) apoE^-/- ^mice in Study 1, and (D) sham-operated controls (0 NX), (E) subtotally nephrectomized and (F) subtotally nephrectomized enalapril-treated (5/6 NX, Enalapril) apoE^-/- ^mice in Study 2. Each bar denotes the maximal thickness of a leaflet. Masson's trichrome stainings from (G) control, (H) subtotally nephrectomized and (I) subtotally nephrectomized enalapril-treated apoE^-/- ^mice show the presence of blue-colored collagen in the leaflets. Note the massive amount of fibrosis in the nephrectomized untreated mice compared with the mild fibrosis of the nephrectomized enalapril-treated mice. Oil Red O stainings from (J) control, (K) subtotally nephrectomized and (L) subtotally nephrectomized enalapril-treated apoE^-/- ^mice. The red lipid staining is present in the atherosclerotic plaques of the aortic wall, but absent from the valve leaflets. Because luminal acellular and unwashable masses are present in (K), the borders of the leaflet are denoted with black arrowheads. Photographed using 10× (A-F) and 20× (G-L) magnification.

Hematoxylin-stained histological sections derived from both studies were photographed with a digital camera (SPOT RT, Diagnostic Instruments Inc., Sterling Heights, MI, USA) attached to a Nikon E600 (Nikon Corp., Tokyo, Japan) light microscope. The sections were coded so that the observer (M.A.S.) was blinded. Six sections from each mouse were photographed at 10× magnification. Maximal leaflet thickness was measured at the nodule of Arantius in the middle of each leaflet (see Figure 3B and Figure 4A–F) with Image-Pro Plus 6.1.0.346 (Media Cybernetics Inc., Silver Spring, MD, USA) software [20]. The thickness of the aortic valve leaflets in each mouse was defined as the average of the three largest leaflet thicknesses measured in the six sections. The total cross-sectional areas of the aortic valve leaflets were measured from three sections per mouse using the Image-Pro Plus software (see Figure 3C). The aortic atherosclerotic *en face *plaque areas have been assessed previously for Study 1 [21] and Study 2 [22].

For Oil Red O staining, the sectioned samples were fixed with paraformaldehyde, washed, and dehydrated in propylene glycol. Thereafter the samples were incubated with O0625 stain (Sigma-Aldrich Corporation, Saint Louis, MO, USA) and differentiated in propylene glycol. Counterstaining was done with Harris's hematoxylin (Merck & Co. Inc., Whitehouse Station, NJ, USA). Masson's trichrome staining was done according to the standard method, modified by Lillie, using a Sigma-Aldrich HT15 kit.

### Statistics

Differences between groups were evaluated by Kruskal-Wallis test and two-group comparisons by Mann-Whitney U test unless specified otherwise. Spearman's (non-parametric data) and Pearson's (parametric data) correlation coefficients were used to assess relationships between continuous variables. Statistical analyses were performed using SPSS 15.0 for Windows (SPSS Inc., Chicago, IL, USA). P-values of < 0.05 were considered statistically significant. Data are expressed as mean ± standard deviation (SD).

## Results

### Effects of uremia and enalapril treatment on plasma biochemistry, body weight, blood pressure, and aortic atherosclerosis

Subtotal nephrectomy (5/6 NX) caused 2.4- to 2.6-fold increases in plasma urea concentrations and affected blood hemoglobin, plasma calcium × phosphate product, plasma cholesterol, and body weight (see Tables 1 and 2). Unilateral nephrectomy (1/2 NX) produced parallel but less pronounced effects (see Table 1). Mean systolic arterial blood pressure was not affected by 5/6 NX. As previously reported [21], 5/6 NX causes a massive increase in aortic atherosclerosis lesion areas. Similar effects were observed in both Study 1 and Study 2. Furthermore, in Study 2, enalapril decreased plasma cholesterol concentration, aortic atherosclerotic lesional area fraction, and systolic arterial blood pressure (see Table 2).

**Table 1:** Effect of chronic renal failure on body weight, blood pressure, blood hemoglobin, plasma indices of uremia, plasma lipids, and aortic atherosclerosis in Study 1.

**Study 1**					
		Unoperated (0 NX)	1/2 NX	5/6 NX	
Variable	Unit	(n = 13)	(n = 18)	(n = 22)	P

Body weight	g	26.4 ± 1.6	27.6 ± 1.5	23.0 ± 3.5^a^	< 0.0001
Mean blood pressure	mmHg	116.6 ± 6.5	113.0 ± 8.2	116.8 ± 14.3	NS
B-hemoglobin	mmol/L	8.38 ± 0.55	8.26 ± 0.26	6.85 ± 0.74^a^	< 0.0001
P-urea	mmol/L	10.6 ± 1.5	12.5 ± 2.1^a^	28.0 ± 9.7^a^	< 0.0001
P-creatinine	mmol/L	0.036 ± 0.005	0.038 ± 0.005	0.051 ± 0.007^a^	< 0.0001
P-phosphate	mmol/L	2.20 ± 0.16	1.89 ± 0.27^a^	2.61 ± 0.72^a^	< 0.0005
P-calcium	mmol/L	2.22 ± 0.12	2.25 ± 0.06	2.52 ± 0.12^a^	< 0.0001
P-Ca × P	mmol^2^/L^2^	4.89 ± 0.43	4.24 ± 0.64^a^	6.56 ± 1.82^a^	< 0.0001
P-cholesterol	mmol/L	13.3 ± 1.9	15.0 ± 3.6	19.3 ± 3.6^a^	< 0.0001
Triglyceride	mmol/L	0.70 ± 0.37	0.58 ± 0.20	0.46 ± 0.14^a^	< 0.01
Aortic plaque area fraction	%	0.048 ± 0.025	0.085 ± 0.038^a^	0.258 ± 0.117^a^	< 0.0001

Fasting blood samples were obtained at the end of the study, i.e. when the mice were 29 weeks of age. Aortic plaque area fraction was determined by the *en face *technique. Values are mean ± SD. P-values were calculated using the Kruskal-Wallis test for differences between three groups. Two-group comparisons were performed using the t-test. Statistically significant difference vs. "Unoperated" group is denoted by superscript a.

**Table 2:** Effects of chronic uremia and enalapril treatment on body weight, blood pressure, blood hemoglobin, plasma indices of uremia, plasma cholesterol, and aortic atherosclerosis in Study 2.

**Study 2**					
		Sham operation (0 NX)	5/6 NX		
				
			No enalapril	Enalapril	
Variable	Unit	(n = 7)	(n = 8)	(n = 13)	P

Body weight	g	32.1 ± 2.0	27.3 ± 2.1^a^	27.4 ± 2.0	< 0.001
Systolic blood pressure	mmHg	112.9 ± 6.1	116.8 ± 4.6	105.2 ± 7.2^b^	< 0.005
B-hemoglobin	mmol/L	9.60 ± 0.83	7.31 ± 0.97^a^	6.99 ± 0.66	< 0.001
P-urea	mmol/L	11.9 ± 1.1	28.6 ± 5.4^a^	26.3 ± 6.2	< 0.001
P-creatinine	mmol/L	0.016 ± 0.002	0.030 ± 0.007^a^	0.025 ± 0.006	< 0.001
P-phosphate	mmol/L	2.47 ± 0.47	2.78 ± 0.65	2.54 ± 0.39	NS
P-calcium	mmol/L	2.70 ± 0.21	2.95 ± 0.41	2.61 ± 0.07	< 0.05
P-Ca × P	mmol^2^/L^2^	6.65 ± 1.45	8.40 ± 3.19	6.63 ± 0.99	NS
P-cholesterol	mmol/L	16.3 ± 2.4	16.6 ± 3.3	13.5 ± 2.9^b^	< 0.05
Aortic plaque area fraction	%	0.10 ± 0.07	0.23 ± 0.08^a^	0.11 ± 0.04^b^	< 0.005

Fasting blood samples obtained at the end of the study, i.e. when the animals were 46 weeks of age. Aortic plaque area fraction was determined by the *en face *technique. Enalapril was administered for 32 weeks with a dose of 2 mg/kg/d starting 4 weeks after the 5/6 nephrectomy. Values are mean ± SD. P-values were calculated using the Kruskal-Wallis test for differences between three groups. Two-group comparisons were performed using the t-test. Statistically significant difference vs. "Sham operation" group is denoted by superscript a. Statistically significant difference vs. "5/6 NX, No enalapril" group is denoted by superscript b.

### Effect of uremia on aortic valve leaflet thickening in unoperated, 1/2 NX, and 5/6 NX apoE^-/- ^mice (Study 1 and Study 2)

**Figure 5:**
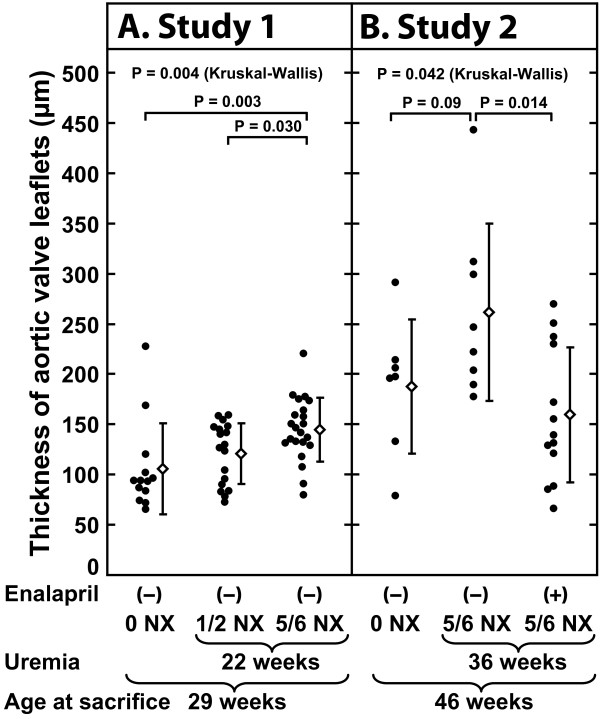
**Effects of uremia and enalapril treatment on the thickness of the aortic valve leaflets in apoE^-/- ^mice**. (A) Effect of 22 weeks of uremia on the thickness of the aortic valve leaflets (Study 1). Three different groups were compared: unoperated (0 NX), unilaterally nephrectomized (1/2 NX) and subtotally nephrectomized (5/6 NX) mice. (B) Effect of 32 weeks of enalapril treatment on the thickness of the aortic valve leaflets induced by uremia lasting for 36 weeks (Study 2). Three groups of mice are shown: sham-operated (0 NX), and subtotally nephrectomized (5/6 NX) mice without and with the drug intervention. Each dot represents a value obtained from an individual mouse. Means are shown as diamonds and standard deviations as vertical bars. Note that, at the time of sacrifice, all the mice in Study 1 were 29 weeks old and all the mice in Study 2 were 46 weeks old.

**Figure 6:**
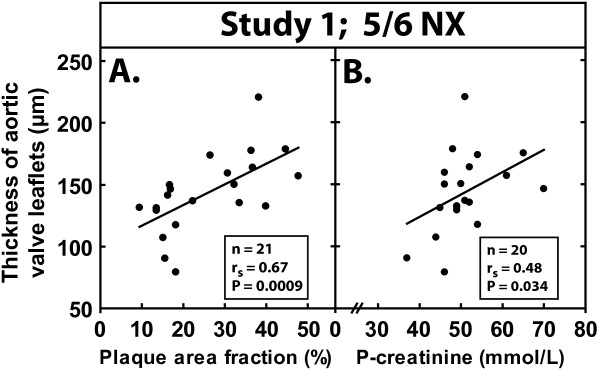
**Relationship between aortic valve leaflet thickening and (A) aortic *en face *plaque area fraction and (B) the level of plasma creatinine in apoE^-/- ^mice with uremia for 22 weeks (Study 1)**. N-values, Spearman's correlation coefficients, and P-values are shown.

After exposure to uremia, the aortic leaflets appeared thickened and the leaflet thickening was diffuse, with maximal thickening located in the central third of the leaflet. To quantitate the effect of renal insufficiency on aortic valve thickening, we measured the maximal aortic valve leaflet thickness at the valve nodule in the nephrectomized and control mice. The analysis of leaflet thickness in the mice exposed to uremia for 22 weeks (Study 1) revealed that the thickness of the aortic valve leaflets was significantly greater in the 5/6 NX mice (145 ± 31 μm) than in the 1/2 NX mice (121 ± 31 μm, P = 0.030) or unoperated mice (106 ± 45 μm, P = 0.003) (see Figure 5A). Also, there was a strong correlation between aortic lesional area and aortic valve leaflet thickness (n = 21, Spearman's r = 0.67, P = 0.0009) in the 5/6 NX mice (see Figure 6A). Moreover, plasma creatinine correlated positively with aortic valve leaflet thickness (n = 20, Spearman's r = 0.48, P = 0.034) in the 5/6 NX mice (see Figure 6B). Analysis of leaflet thickness in the mice exposed to uremia for 36 weeks (Study 2) showed a trend of uremia-induced aortic valve thickening, although the result was not statistically significant due to the small number of mice. There was a strong linear correlation between the thickness of the aortic valve leaflets and the total cross-sectional area (P = 0.00000037) (see Figure 3).

### Effect of enalapril on aortic valve leaflet thickening in 5/6 NX apoE^-/- ^mice (Study 2)

After 36 weeks of uremia, we examined the effect of ACE inhibition on maximal valve thickness in the 5/6 NX apoE^-/- ^mice. The 5/6 NX mice that had been treated with 2 mg/kg/d enalapril for 32 weeks had significantly thinner aortic valve leaflets when compared with the 5/6 NX mice that had not been treated with enalapril (159 ± 67 μm vs. 261 ± 88 μm, P = 0.014) (see Figure 5B).

### Histopathologic findings (Study 2)

To examine for the presence of fibrous and neutral lipid components in the valves, the sections were stained with Massons's trichrome and Oil Red O, respectively. As shown in Figure 4G–I, the valvular leaflets in all three groups contained collagen. In the thickened leaflets, there was not only more collagen, but the collagen bundles formed dense deposits, reflecting fibrous thickening of the valves. The leaflets of the 5/6 NX mice treated with enalapril appeared to contain less collagen than the leaflets of their untreated littermates, and the collagen bundles were arranged in a looser fashion.

Since all the animals in this animal model of aortic valvular thickening were hyperlipidemic apoE^-/- ^mice, their aortas contained atherosclerotic plaques (see Tables 1 and 2). As expected, also the aortic wall in the aortic root region contained Oil Red O-positive intimal thickenings (see Figure 4J–L). In sharp contrast, Oil Red O staining was negative in the leaflets, demonstrating that, despite the presence of marked hyperlipidemia and uremia, the valvular pathology did not involve deposition of neutral lipids, i.e. cholesteryl esters or triglycerides, in the leaflets.

## Discussion

The present study demonstrates that uremia accelerates aortic valve thickening in genetically hyperlipidemic mice. Importantly, aortic valve thickening in the nephrectomized mice was inhibited by enalapril, suggesting that uremia induces aortic valve sclerosis or stenosis via Ang II-mediated mechanisms. These findings emphasize the significance of local RAS in the pathobiology of aortic valve disease and raise pharmacological prevention of RAS actions as a potential treatment option in this disease.

Indeed, recent investigations have portrayed the possibility that activation of RAS promotes AS progression and, thus, inhibition of its actions may be beneficial in this disease [23]. ACE is locally produced in aortic valves and its enzymatic activity is augmented in stenotic leaflets [13]. Furthermore, Ang II and profibrotic Ang II type 1 receptors (AT-1Rs) are present in diseased valves and colocalize with ACE in valvular lesions [12,13]. In addition to ACE, two alternative Ang II-forming enzymes, namely chymase and cathepsin G, are upregulated in stenotic valves, further enhancing the Ang II-forming potential of the affected valves [10,13]. Accordingly, Ang II, as a powerful proinflammatory and profibrotic mediator [24], may participate in the pathogenesis of AS both in subjects with preserved renal function and, as suggested by the present results, also in uremic patients. Preliminary support for the benefit of ACE inhibitors in AS patients originates from small clinical studies in which ACE inhibitors have been shown to be well tolerated in AS patients and to even improve their hemodynamic status and exercise tolerance [25-27]. Moreover, in a recent retrospective study by O'Brien and co-workers, the use of ACE inhibitors was associated with slower progression of aortic valve calcification [28]. In contrast, Rosenhek and co-workers were not able to demonstrate any beneficial effects of ACE inhibitors on the hemodynamic progression of AS [29]. Considering the retrospective nature and small sample sizes of these trials, additional research in this area is indispensable.

In epidemiological studies, the prevalence of aortic valve abnormalities, including valve sclerosis, calcification, and stenosis, is increased in patients with renal failure [30-32]. Moreover, in patients with renal insufficiency, the rate of AS progression is higher than in those with normal renal function [3,33]. In view of these observations, chronic uremia and its consequences seem to exert detrimental effects on the aortic valve leaflets. Several uremia-related mechanisms may contribute to the thickening of the aortic valve leaflets. Renal failure is associated with abnormal calcium-phosphate homeostasis, including hyperphosphatemia, a high serum calcium phosphate product, and secondary hyperpharathyroidism, which may lead to metastatic calcification of tissues, including the aortic valve leaflets [3,34]. Furthermore, chronic renal insufficiency leads to activation of RAS [35], which constitutes a risk factor of cardiovascular calcification in humans. Bilateral total nephrectomy (6/6 NX) in rats has been shown to cause plasma renin to fall below the limits of detection within 24–48 hours, thus effectively eliminating circulating RAS [36]. Because of the massive reduction in kidney tissue, it is also likely that the levels of circulating renin were decreased in the 5/6 nephrectomized mice in the present study, as well. However, ACE is produced mainly in the vascular bed of the lungs [37]. According to our previously published results, 5/6 nephrectomy increases plasma ACE activity by 36 percent [22]. Thus, inhibition of ACE should be an effective and appropriate treatment strategy in the present animal model.

In addition to the mouse models described above, several rabbit models of AS have been introduced [14-17]. Rajamannan and co-workers described a hypercholesterolemic rabbit model in which hypercholesterolemia resulted in aortic valve thickening, atherosclerotic changes, and bone matrix production in the valves, and these adverse processes were prevented by atorvastatin [15,38,39]. In contrast, in the rabbit model described by Drolet and co-workers, hypercholesterolemia alone did not cause any significant functional aortic valve abnormality when assessed by echocardiography [16]. Instead, hypercholesterolemia combined with toxic doses of vitamin D resulted in decreased aortic valve area (AVA) and increased transvalvular gradients in these animals [16]. In a recent rabbit model of AS, hypercholesterolemia induced fatty deposition, macrophage accumulation, and osteopontin expression in the aortic valves, and these atherosclerotic changes and expression of osteoblastic factors were attenuated by the AT-1R antagonist olmesartan [17]. Furthermore, dietary hypercholesterolemia disrupted endothelial integrity in lesion-prone areas of the valves, whereas preservation of the endothelium was attained with olmesartan [17]. These observations indicate that aortic valve lesion development caused by hypercholesterolemia is not only related to cholesterol accumulation. Instead, activation of RAS appears as a key element in lesion development, including both induction of inflammation and osteoblastic transdifferentiation of valvular cells.

The exact molecular mechanism by which enalapril prevents aortic valve leaflet thickening remains unknown. Besides being capable of inhibiting ACE enzyme locally, enalapril is also able to exert multiple systemic effects. The only statistically significant changes in metabolic indices due to the enalapril treatment were lowering of systolic blood pressure and plasma total cholesterol. Interestingly, a recent study in humans has shown that ACE inhibitors may have a beneficial effect on the lipid profile [40]. However, the very recently published SEAS study has clearly shown that even a long-lasting lowering of the plasma cholesterol level by 60 percent fails to affect the progression of aortic stenosis [41]. Importantly, enalapril may contribute to the inhibition of valvular thickening also by lowering systolic blood pressure. Excluding this possibility remains a challenge for future studies.

No animal model for uremia-induced aortic valve thickening has been published previously. In the present study, the increased prevalence of aortic valve leaflet thickening among the hyperlipidemic mice with unilateral nephrectomy, and consequently mild renal impairment, suggests that even mild renal insufficiency promotes aortic valve leaflet thickening and eventually may increase the risk of aortic stenosis in hyperlipidemic mice. Importantly, in the 5/6 nephrectomized uremic mice, we noted that 22 weeks of uremia (Study 1) induced less thickening of the leaflets than did 36 weeks of uremia (Study 2), providing additional support for the idea that uremia induces gradual valve thickening in this hyperlipidemic mouse model. Furthermore, a strong correlation appeared between aortic atherosclerotic lesional area and aortic valve thickness (see Figure 6A). This is of interest, since epidemiological studies have shown that AS and arterial atherosclerosis share common risk factors, including uremia, hypercholesterolemia, hypertension, smoking, diabetes, age, and male gender [5-7]. Furthermore, coronary artery disease coexists in approximately 50% of patients with AS [42], and even patients with aortic valve sclerosis, an early stage of AS, possess an elevated risk of myocardial infarction and cardiovascular death [43]. Also, there was a significant correlation between plasma creatinine level and aortic valve thickness (see Figure 6B). Creatinine, being the marker of renal dysfunction, suggests that uremia is an important etiological factor of aortic valve thickening in this particular model.

The following facts could be considered as limitations of this study. The blood pressure-lowering effect of enalapril may have contributed to the decreased thickening of the valve leaflets. Although we demonstrate here that enalapril prevents aortic valve thickening in nephrectomized apoE^-/- ^mice, we do not provide evidence concerning the efficacy of enalapril in other mouse models of AS. Appropriate animal models of AS that closely mimic the disease etiology and manifestations in humans are difficult to establish, and the utilized models have several limitations. In this sense, the present model of uremic apoE^-/- ^mice has the advantage of sharing the same etiology with human AS in patients whose aortic valve disease is associated with chronic renal failure and hypercholesterolemia. Previously, several mouse models of AS have been tested, i.e. senile apoE^-/- ^mice [18], LDLr^-/- ^apoB^100/100 ^mice [19], nephrectomized fetuin-A-knockout mice with a high phosphate diet [44], and wild-type mice fed a high fat/high carbohydrate diet [20]. The general problem in mouse models is the difficulty in assessing functional impairment in the valve, i.e. challenging echocardiography. In the present study, aortic valve echocardiography was not part of the study design, as the focus was placed on early histopathological changes in the valves, i.e. at a stage in which no significant functional impairment of the valves was to be expected (22 weeks in Study 1 and 36 weeks in Study 2). Indeed, previous models have described functional changes only in older animals, and echocardiac abnormalities were not evident in the apoE^-/- ^mice studied by Tanaka and co-workers until 80–120 weeks of age [18]. Finally, the duration of uremia was different in the two studies (22 weeks in Study 1 vs. 36 weeks in Study 2). However, aortic valve thickening was observed in both studies.

## Conclusion

The present study demonstrates a novel mouse model of aortic valve thickening, the uremic apoE^-/- ^mouse, and reveals a potential treatment of uremia-induced valvular pathology by an ACE inhibitor. Although the mice were severely hyperlipidemic and atherosclerotic, aortic valve thickening presented itself as a fibrotic disease, not a lipid disease. As aortic valve thickening represents an early stage of AS, this animal constitutes a model for exploring the development and pathogenesis of AS and its treatment by pharmacological therapy, particularly with drugs that inhibit the production and pathological effects of Ang II.

## Competing interests

The authors declare that they have no competing interests.

## Authors' contributions

MAS carried out the staining, aortic valve leaflet thickness measurement, statistical analysis and drafting of the manuscript. TXP and SB raised the mice and performed the biochemical analyses. LBN participated in the design of the study. MIM conceived the study and participated in its design, coordination, and in the drafting of the manuscript. SH helped to draft the manuscript. PTK contributed to the design of the study, interpretation of the data, drafting and revising of the manuscript. All the authors read and approved the final manuscript.

## Pre-publication history

The pre-publication history for this paper can be accessed here:



## References

[B1] Foley RN, Parfrey PS, Sarnak MJ (1998). Clinical epidemiology of cardiovascular disease in chronic renal disease. Am J Kidney Dis.

[B2] Maher ER, Young G, Smyth-Walsh B, Pugh S, Curtis JR (1987). Aortic and mitral valve calcification in patients with end-stage renal disease. Lancet.

[B3] Perkovic V, Hunt D, Griffin SV, du Plessis M, Becker GJ (2003). Accelerated progression of calcific aortic stenosis in dialysis patients. Nephron Clin Pract.

[B4] Ohara T, Hashimoto Y, Matsumura A, Suzuki M, Isobe M (2005). Accelerated progression and morbidity in patients with aortic stenosis on chronic dialysis. Circ J.

[B5] Stewart BF, Siscovick D, Lind BK, Gardin JM, Gottdiener JS, Smith VE, Kitzman DW, Otto CM (1997). Clinical factors associated with calcific aortic valve disease. Cardiovascular Health Study. J Am Coll Cardiol.

[B6] Lindroos M, Kupari M, Valvanne J, Strandberg T, Heikkila J, Tilvis R (1994). Factors associated with calcific aortic valve degeneration in the elderly. Eur Heart J.

[B7] Deutscher S, Rockette HE, Krishnaswami V (1984). Diabetes and hypercholesterolemia among patients with calcific aortic stenosis. J Chronic Dis.

[B8] Otto CM, Kuusisto J, Reichenbach DD, Gown AM, O'Brien KD (1994). Characterization of the early lesion of 'degenerative' valvular aortic stenosis. Histological and immunohistochemical studies. Circulation.

[B9] Mohler ER, Gannon F, Reynolds C, Zimmerman R, Keane MG, Kaplan FS (2001). Bone formation and inflammation in cardiac valves. Circulation.

[B10] Helske S, Syvaranta S, Kupari M, Lappalainen J, Laine M, Lommi J, Turto H, Mayranpaa M, Werkkala K, Kovanen PT, Lindstedt KA (2006). Possible role for mast cell-derived cathepsin G in the adverse remodelling of stenotic aortic valves. Eur Heart J.

[B11] Helske S, Syvaranta S, Lindstedt KA, Lappalainen J, Oorni K, Mayranpaa MI, Lommi J, Turto H, Werkkala K, Kupari M, Kovanen PT (2006). Increased expression of elastolytic cathepsins S, K, and V and their inhibitor cystatin C in stenotic aortic valves. Arterioscler Thromb Vasc Biol.

[B12] O'Brien KD, Shavelle DM, Caulfield MT, McDonald TO, Olin-Lewis K, Otto CM, Probstfield JL (2002). Association of angiotensin-converting enzyme with low-density lipoprotein in aortic valvular lesions and in human plasma. Circulation.

[B13] Helske S, Lindstedt KA, Laine M, Mayranpaa M, Werkkala K, Lommi J, Turto H, Kupari M, Kovanen PT (2004). Induction of local angiotensin II-producing systems in stenotic aortic valves. J Am Coll Cardiol.

[B14] Kwon HM, Lee BK, Kim D, Hong BK, Byun KH, Kna JS, Kim IJ, Oh SH, Kim HS (1998). Experimental hypercholesterolemia induces ultrastructural changes in the elastic laminae of rabbit aortic valve. Yonsei Med J.

[B15] Rajamannan NM, Subramaniam M, Springett M, Sebo TC, Niekrasz M, McConnell JP, Singh RJ, Stone NJ, Bonow RO, Spelsberg TC (2002). Atorvastatin inhibits hypercholesterolemia-induced cellular proliferation and bone matrix production in the rabbit aortic valve. Circulation.

[B16] Drolet MC, Arsenault M, Couet J (2003). Experimental aortic valve stenosis in rabbits. J Am Coll Cardiol.

[B17] Arishiro K, Hoshiga M, Negoro N, Jin D, Takai S, Miyazaki M, Ishihara T, Hanafusa T (2007). Angiotensin receptor-1 blocker inhibits atherosclerotic changes and endothelial disruption of the aortic valve in hypercholesterolemic rabbits. J Am Coll Cardiol.

[B18] Tanaka K, Sata M, Fukuda D, Suematsu Y, Motomura N, Takamoto S, Hirata Y, Nagai R (2005). Age-associated aortic stenosis in apolipoprotein E-deficient mice. J Am Coll Cardiol.

[B19] Weiss RM, Ohashi M, Miller JD, Young SG, Heistad DD (2006). Calcific aortic valve stenosis in old hypercholesterolemic mice. Circulation.

[B20] Drolet MC, Roussel E, Deshaies Y, Couet J, Arsenault M (2006). A high fat/high carbohydrate diet induces aortic valve disease in C57BL/6J mice. J Am Coll Cardiol.

[B21] Bro S, Bentzon JF, Falk E, Andersen CB, Olgaard K, Nielsen LB (2003). Chronic renal failure accelerates atherogenesis in apolipoprotein E-deficient mice. J Am Soc Nephrol.

[B22] Bro S, Binder CJ, Witztum JL, Olgaard K, Nielsen LB (2007). Inhibition of the renin-angiotensin system abolishes the proatherogenic effect of uremia in apolipoprotein E-deficient mice. Arterioscler Thromb Vasc Biol.

[B23] Routledge HC, Townend JN (2001). ACE inhibition in aortic stenosis: dangerous medicine or golden opportunity?. J Hum Hypertens.

[B24] Mehta PK, Griendling KK (2007). Angiotensin II cell signaling: physiological and pathological effects in the cardiovascular system. Am J Physiol Cell Physiol.

[B25] O'Brien KD, Zhao XQ, Shavelle DM, Caulfield MT, Letterer RA, Kapadia SR, Probstfield JL, Otto CM (2004). Hemodynamic effects of the angiotensin-converting enzyme inhibitor, ramipril, in patients with mild to moderate aortic stenosis and preserved left ventricular function. J Investig Med.

[B26] Chockalingam A, Venkatesan S, Subramaniam T, Jagannathan V, Elangovan S, Alagesan R, Gnanavelu G, Dorairajan S, Krishna BP, Chockalingam V, Symptomatic Cardiac Obstruction-Pilot Study of Enalapril in Aortic Stenosis (2004). Safety and efficacy of angiotensin-converting enzyme inhibitors in symptomatic severe aortic stenosis: Symptomatic Cardiac Obstruction-Pilot Study of Enalapril in Aortic Stenosis (SCOPE-AS). Am Heart J.

[B27] Jimenez-Candil J, Bermejo J, Yotti R, Cortina C, Moreno M, Cantalapiedra JL, Garcia-Fernandez MA (2005). Effects of angiotensin converting enzyme inhibitors in hypertensive patients with aortic valve stenosis: a drug withdrawal study. Heart.

[B28] O'Brien KD, Probstfield JL, Caulfield MT, Nasir K, Takasu J, Shavelle DM, Wu AH, Zhao XQ, Budoff MJ (2005). Angiotensin-converting enzyme inhibitors and change in aortic valve calcium. Arch Intern Med.

[B29] Rosenhek R, Rader F, Loho N, Gabriel H, Heger M, Klaar U, Schemper M, Binder T, Maurer G, Baumgartner H (2004). Statins but not angiotensin-converting enzyme inhibitors delay progression of aortic stenosis. Circulation.

[B30] Maher ER, Pazianas M, Curtis JR (1987). Calcific aortic stenosis: a complication of chronic uraemia. Nephron.

[B31] Baglin A, Hanslik T, Vaillant JN, Boulard JC, Moulonguet-Doleris L, Prinseau J (1997). Severe valvular heart disease in patients on chronic dialysis. A five-year multicenter French survey. Ann Med Interne (Paris).

[B32] Raine AE (1994). Acquired aortic stenosis in dialysis patients. Nephron.

[B33] Urena P, Malergue MC, Goldfarb B, Prieur P, Guedon-Rapoud C, Petrover M (1999). Evolutive aortic stenosis in hemodialysis patients: analysis of risk factors. Nephrologie.

[B34] London GM, Pannier B, Marchais SJ, Guerin AP (2000). Calcification of the aortic valve in the dialyzed patient. J Am Soc Nephrol.

[B35] Vlahakos DV, Hahalis G, Vassilakos P, Marathias KP, Geroulanos S (1997). Relationship between left ventricular hypertrophy and plasma renin activity in chronic hemodialysis patients. J Am Soc Nephrol.

[B36] Pedersen TF, Nielsen AH, Strandgaard S, Paulson OB (2002). Nephrectomy and peritoneal dialysis eliminates circulating renin and controls uraemia in the rat. J Renin Angiotensin Aldosterone Syst.

[B37] Campbell DJ (1987). Circulating and tissue angiotensin systems. J Clin Invest.

[B38] Rajamannan NM, Subramaniam M, Caira F, Stock SR, Spelsberg TC (2005). Atorvastatin inhibits hypercholesterolemia-induced calcification in the aortic valves via the Lrp5 receptor pathway. Circulation.

[B39] Rajamannan NM, Subramaniam M, Stock SR, Stone NJ, Springett M, Ignatiev KI, McConnell JP, Singh RJ, Bonow RO, Spelsberg TC (2005). Atorvastatin inhibits calcification and enhances nitric oxide synthase production in the hypercholesterolaemic aortic valve. Heart.

[B40] Nandeesha H, Pavithran P, Madanmohan T (2008). Effect of Antihypertensive Therapy on Serum Lipids in Newly Diagnosed Essential Hypertensive Men. Angiology.

[B41] Rossebo AB, Pedersen TR, Boman K, Brudi P, Chambers JB, Egstrup K, Gerdts E, Gohlke-Barwolf C, Holme I, Kesaniemi YA, Malbecq W, Nienaber CA, Ray S, Skjaerpe T, Wachtell K, Willenheimer R, SEAS Investigators (2008). Intensive lipid lowering with simvastatin and ezetimibe in aortic stenosis. N Engl J Med.

[B42] Otto CM, Burwash IG, Legget ME, Munt BI, Fujioka M, Healy NL, Kraft CD, Miyake-Hull CY, Schwaegler RG (1997). Prospective study of asymptomatic valvular aortic stenosis. Clinical, echocardiographic, and exercise predictors of outcome. Circulation.

[B43] Otto CM, Lind BK, Kitzman DW, Gersh BJ, Siscovick DS (1999). Association of aortic-valve sclerosis with cardiovascular mortality and morbidity in the elderly. N Engl J Med.

[B44] Westenfeld R, Schafer C, Smeets R, Brandenburg VM, Floege J, Ketteler M, Jahnen-Dechent W (2007). Fetuin-A (AHSG) prevents extraosseous calcification induced by uraemia and phosphate challenge in mice. Nephrol Dial Transplant.

